# The Human Microbiota: The Rise of an “Empire”

**DOI:** 10.5041/RMMJ.10202

**Published:** 2015-04-29

**Authors:** Gidon Berger, Roni Bitterman, Zaher S. Azzam

**Affiliations:** 1 Department of Internal Medicine B, Rambam Health Care Campus, Haifa, Israel;; 2 Ruth & Bruce Rappaport Faculty of Medicine, Technion, Israel Institute of Technology, Haifa, Israel

**Keywords:** 16S ribosomal RNA, human microbiota, next-generation sequencing

## Abstract

The human body hosts rich and diverse microbial communities. Our microbiota affects the normal human physiology, and compositional changes might alter host homeostasis and, therefore, disease risk. The microbial community structure may sometimes occupy discrete configurations and under certain circumstances vary continuously. The ability to characterize accurately the ecology of human-associated microbial communities became possible by advances in deep sequencing and bioinformatics analyses.

## THE NORMAL HUMAN MICROBIOTA

The American microbiologist and biophysicist, Carl Woese, defined a three-domain system of taxonomy in which a domain (also empire) is the highest taxonomic rank of organisms. According to the Woese system, introduced in 1990, the tree of life consists of three domains: Archaea, Bacteria, and Eukarya.^1^ Many members of the first and second domains live a life of convenience within us. The human body hosts complex microbial communities whose combined membership outnumbers our own cells by at least a factor of 10.^2^,^3^ In order to characterize the ecology of human-associated microbial communities, the National Institutes of Health launched in 2007 the Human Microbiome Project (HMP). The findings of this sentinel study were published in 2012. Briefly, a total of 4,788 specimens from 18 female body habitats and 15 male body habitats representing five major body areas (oral cavity and oropharynx, skin, nostrils, gastrointestinal tract, and vagina) were collected from 242 healthy adults. These samples were subjected to 16S ribosomal RNA (16S rRNA) gene pyrosequencing, and a subset were shotgun-sequenced for metagenomics. Rich communities in each of the body’s habitats were found with strong niche specialization both within and among individuals. Interestingly, oral (considered part of the upper respiratory tract) and stool communities were especially diverse in terms of community membership.^4^ It was shown that, within habitats, interpersonal variability is high, whereas individuals exhibited minimal temporal variability.^4^,^5^

Analyses of the taxonomic diversity associated with the human microbiota (the collection of micro-organisms that are present in a community from a defined body habitat) became an area of great importance. The study of the nature and extent of the commonly shared taxa (“core”), versus those less prevalent, establishes a baseline for comparing healthy and diseased groups by quantifying the variation among people, across body habitats, and over time. The HMP has provided an unprecedented opportunity to examine and define better what constitutes the taxonomic core within and across body habitats and individuals. A two-parameter (taxonomic ubiquity and abundance) model was introduced by Li et al. to identify quantitatively the core taxonomic members of each body habitat’s microbiota across the healthy cohort.^6^ Although many microbes were shared at low abundance, they exhibited a relatively continuous spread in both their abundance and ubiquity, as opposed to a more discretized separation. The numbers of core taxa members in the body regions are comparatively small and stable, reflecting the relatively high, but conserved, interpersonal variability within the cohort. Core sizes increased across the body regions in the order of: vagina, skin, stool, and oral cavity. A number of “minor” oral taxonomic cores were also identified by their majority presence across the cohort, but with relatively low and stable abundances. A method for quantifying the difference between two cohorts was introduced and applied to samples collected on a second visit, revealing that, over time, the oral, skin, and stool body regions tended to be more transient in their taxonomic structure than the vaginal body region.^6^

The human microbiota harbors thousands (and perhaps many more) of bacterial taxa. Over time the full picture is revealed and novel bacterial taxa are being identified. Wylie et al. assessed metagenomic data generated by the HMP to determine if novel taxa remain to be discovered in stool samples from healthy individuals.^7^ They discovered several low-abundance, novel bacterial taxa, which span three major phyla in the bacterial tree of life. They determined that these taxa are present in a larger set of HMP subjects and are found in two sampling sites (Houston, Texas and St. Louis, Missouri, USA). The majority of novel sequences are related to the recently discovered genus *Barnesiella*, further encouraging efforts to characterize the members of this genus and to study their roles in the microbial communities of the gut. Understanding the effects of less-abundant bacteria is important as we seek to understand the complex gut microbiome in healthy individuals and link changes in the microbiome to disease.^7^

## THE ROLE OF MICROBIOTA IN ILLNESS

Kluyver et al. stated that “the only truly scientific foundation of classification is to be found in appreciation of the available facts from a phylogenetic point of view. Only in this way can the natural interrelationships [among organisms] be properly understood.”^8^

Modern medical microbiology focused on certain pathogenic bacteria, while the population of microbes in and on the human body was mostly considered to be vast and largely unknowable. It was referred to as “the normal flora,” the collection of “plants” living with us humans, and was treated as a black box. By and large, considering the overall scope of medical research, the microbiome was a backwater, the field of some highly specialized scientists and a few generalist pioneers.^9^,^10^ But then, things began to change, and this domain has recently emerged as an important factor in human physiology and disease.

The dominant forms of interactions of humans and micro-organisms are commensal relationship and symbiotic relationship. Together, our ∼100 trillion microbial symbionts endow us with crucial traits: the human microbiota facilitates the extraction of energy from food, provides accessory growth factors, promotes post-natal terminal differentiation of mucosal structure and function, stimulates both the innate and adaptive immune systems, and provides “colonization resistance” against pathogen invasion.^11^–^14^ If our microbiota affects human physiology, it should be no surprise that compositional changes might alter host homeostasis and, therefore, disease risk. Indeed, analysis of the human microbiota implicates global alteration of microbial communities in a wide spectrum of human diseases such as asthma,^15^ obesity,^16^,^17^ bacterial vaginosis,^18^ and inflammatory bowel disease (IBD).^19^,^20^ Regarding the last-mentioned, for instance, it is now generally accepted that altered composition and function of the commensal enteric bacteria provide the constant antigenic stimulation which, in turn, continuously activates pathogenic T cells with resultant chronic intestinal injury.^20^ The characteristics of the dysbiotic microbiota associated with IBD have been highly reproducible, including an enrichment of bacterial taxa belonging to the *Proteobacteria* and *Actinobacteria* phyla, a decrease in representation of *Firmicutes*, and a reduction in microbial richness, the last-mentioned being an indication that there are fewer microbial species in total.^21^ Although it is relatively difficult to establish a causal association between the microbiome and many of the chronic diseases described above, establishing a causal association with acute onset of infectious diseases such as *Clostridium difficile* infection (CDI) is easier. Actually, CDI is the only disease process in which it was demonstrated that the dysbiotic microbiota plays a role in disease pathogenesis and in which restoration of the normal healthy microbiota is an effective therapy. Consumption of antibiotics dramatically, but transiently, alters the composition of the gut microbiota, providing a niche in which *C. difficile* can expand.^22^

## THE EVOLUTION OF THE MICROBIOTA

As was shown in the HMP, the bacterial diversity in the human body is striking in its richness of distinct species and strains; however, it is noteworthy that a limited number of phyla are commonly found in indigenous microbial communities. Only four of the more than 50 bacterial phyla that have been identified in the environment (Firmicutes, Bacteroidetes, Actinobacteria, and Proteobacteria) dominate human mucosal and cutaneous habitats, which suggests that strong selective forces have limited diversity over at least hundreds of thousands of years of co-evolution.^23^–^25^ Despite this stereotypical assembly process, each individual in a single mammalian species, including *Homo sapiens*, has a virtually unique microbiota.^26^,^27^ The composition of the indigenous microbiota evolved over millions of years in a generally orderly manner in response to diet and other environmental factors and is also influenced by diverse human genetic backgrounds. However, beginning in the nineteenth century and accelerating in the twentieth century, there have been dramatic changes in human ecology, including cleaner water, smaller families, an increased number of Caesarian sections, increased use of pre-term antibiotics, lower rates of breastfeeding, and more than 60 years of widespread antibiotic use, particularly in young children. And as human ecology changes, so does our microbiota. The permanent and widespread change in our microecology, in analogy to our altered macroecology, is referred to as the “disappearing microbiota” hypothesis.^28^,^29^ The following two observations describe disappearing bacteria and the consequences of loss. Although *Helicobacter pylori* was once present in almost every adult human, the bacterium is now rapidly disappearing from human populations owing to changes in sanitation, demographics, and antibiotic usage. Today, fewer than 10% of children in the USA harbor this bacterium in their stomach. *Helicobacter pylori* modulates immunological, endocrine, and physiological functions in the stomach.^30^ The biological costs of carrying *H. pylori* include peptic ulcers and adenocarcinoma of the distal stomach. Conversely, certain strains also protect against gastroesophageal reflux disease (GERD) and its consequences, including esophageal adenocarcinoma, owing in part to their effects on gastric acid secretion.^28^ These observations are consistent with the rise of these diseases wherever *H. pylori* is disappearing. *Streptococcus pneumoniae* (known as the pneumococcus) is an important human pathogen, causing pneumococcal pneumonia, infections of the upper respiratory tract and its appendages, and occasionally lethal diseases such as meningitis and endocarditis.^31^ However, pneumococci are carried by healthy persons in the nasopharynx, often for months, and are part of the consortia of micro-organisms inhabiting this niche. The clinical significance *of S. pneumoniae* pushed for vaccine development. These vaccines are effective and have reduced the incidence of serious pneumococcal infections in high-risk populations.^32^ Immunization not only protects against disease but also prevents colonization by those pneumococci with the capsule types that are present in the vaccine.^33^ Except for the predicted consequences of replacement with non-vaccine serotypes of *S. pneumoniae*,^32^,^34^,^35^ replacement with an unanticipated violent organism, *Staphylococcus aureus*, has occurred.^34^,^35^ These observations provide definitive examples of diseases caused by changes in the human microbiota. Except for these global phenomena, alteration in our microecology and consequently our health might occur on an individual basis, as each individual’s microbiota is subjected during the life to a wide spectrum of specific host-dependent factors such as smoking,^36^ vaccinations, and antibiotic use.

## MICROBIOTA RESEARCH METHODS

The ability to characterize accurately the complex structure and rich composition of these microbial communities became possible by advances in deep sequencing and bioinformatics analyses. Unlike conventional methods that can detect only a single microbe in a sample, the high-throughput, massively parallel, next-generation sequencing allows identification of almost the entire microbiota present in a sample. With 16S rRNA sequencing, the final data set consists of thousands to millions of sequences from a segment of the 16S rRNA gene. Each sequence is taken to represent an individual micro-organism, and the collection of sequences is taken to be representative of the community as a whole in terms of both types of organisms present and their relative abundance to one another. The bacterial 16S rRNA genes generally contain nine “hypervariable regions” that demonstrate considerable sequence diversity among different bacterial species and can therefore be used for species identification. Hyper-variable regions are flanked by conserved stretches in most bacteria, enabling PCR amplification of target sequences using universal primers.

Furthermore, by using even newer technologies capable of sequencing billions of DNA base pairs in a single run at an affordable cost, shotgun meta-genomic sequencing can be performed in which community DNA is sequenced in totality, permitting not only an evaluation of microbial community structure but also allowing an evaluation of the genomic representation of the community. The latter can be used to help understand the functions encoded by the genomes of the microbiota. Shotgun metagenomic sequencing also can be used to characterize the abundance of viruses, or the virome, biological entities that lack ribosomal genes yet are among the most abundant organisms in the biosphere.^37^

In conclusion, the human body harbors thousands of different bacterial taxa. The importance of this ecosystem is immense as analysis of the human microbiota implicates global alteration of microbial communities in a wide spectrum of human diseases.

## Abbreviations:

CDI: *Clostridium difficile* infection;
HMP: Human Microbiome Project;
IBD: inflammatory bowel disease.
